# Screening for tuberculosis in an urban shelter for homeless in Switzerland: a prospective study

**DOI:** 10.1186/s12879-017-2449-y

**Published:** 2017-05-16

**Authors:** Jean-Paul Janssens, Timothee Wuillemin, Dan Adler, Yves Jackson

**Affiliations:** 1 Division of Pneumology, Geneva University Hospitals, Geneva, Switzerland; 2 Division of Primary Care Medicine, Geneva University Hospitals, Rue Gabrielle-Perret-Gentil 6, 1211 Geneva, Switzerland; 3 Insitute of Global Health, Geneva University, Geneva, Switzerland

**Keywords:** Tuberculosis, Screening, Homeless, Control, Switzerland

## Abstract

**Background:**

Whereas high risk groups such as asylum seekers are systematically screened for active tuberculosis (TB) upon entry in Switzerland, this strategy does not apply to homeless persons despite a reported high risk. Geneva health and social authorities implemented an intersectoral project to screen for active TB in homeless persons. We aimed to assess acceptability of this program and prevalence of active TB in this group.

**Methods:**

This prospective study targeted all homeless adults registering for shelter accommodation in Geneva during winter 2015. Applicants were proposed a questionnaire-based screening (www.tb-screen.ch) exploring epidemiological and clinical risk factors for active TB. Participants with a positive score underwent diagnostic procedures at Geneva University Hospital. Enhanced TB surveillance targeting homeless persons in the community was continued 3 months after the study termination.

**Results:**

Overall, 726/832 (87.3%) homeless persons accepted the screening procedure. Most were young male migrants without access to care in Switzerland. Male gender (adjusted OR: 2.14; 95% confidence interval: 1.27–3.62), age below 25 years (aOR: 4.16; 95% CI: 1.27–13.64) and short duration of homelessness (aOR: 1.75; 95% CI: 1.06–2.87) were predictors of acceptance. Thirty (4.1%) had positive screening scores but none of the 24 who underwent further testing had active TB. Post-study surveillance did not identify any incident case in Geneva.

**Conclusions:**

Active TB screening targeting highly mobile homeless persons in shelters was well accepted and feasible. The participants’ sociodemographic profile highlighted the heterogeneity of homeless groups in Europe and the null TB prevalence the variability of their active TB risks. These findings underline the feasibility of health programs targeting this hard to reach group and the need for close monitoring of this social group considering the rapid changes in international mobility patterns to tailor preventive and screening strategies to the local context.

## Background

Homelessness, a global and mostly urban phenomenon, encompasses a spectrum of social situations ranging from inadequate accommodation to the complete lack of housing [1]. In the European Union, it affects an estimated 410′000 people each night and 4.1 million annually [2]. There is no data currently available about the situation in Switzerland.

Homelessness exposes to harsh physical and social environments with a negative impact on health. In Europe, mortality in homeless persons is seven times higher than in the general population and morbidity, both somatic and psychiatric, is also significantly increased [3, 4]. It is associated with a substantial risk of communicable diseases, notably tuberculosis (TB) [5, 6]. In Western Europe, TB prevalence in homeless persons is between 1 and 2% for active and up to 45% for latent infection [5, 7–9]. This prevalence, considerably higher than in the general population, underlines the public health importance of developing control programs targeting this group. To enhance TB control program effectiveness, guidelines recommend using active decentralized (ie. outside healthcare settings) case-finding strategies in cooperation with community stakeholders and local social services [10, 11]. While screening and treatment is cost effective (US$ 32′000 per QALY) compared to passive strategies among groups with a TB prevalence of 250/100′000 or higher in Europe, low awareness of TB, limited participation in programs, and frequent drop out along diagnostic and therapeutic procedures frequently limit effectiveness of control programs [5, 12, 13]. Various screening modalities have been tested and there is still debate over the optimal strategy [14]. Although there is no gold standard methods for screening active tuberculosis in underserved populations, active case-finding strategies usually rely on chest-X-ray and sputum analysis, sometimes combined with symptoms score. In registry-based studies, chest X-ray provided a limited sensitivity of 42% (95% confidence interval: 28–58%) [15].

In Switzerland, active TB incidence is 7.4/100′000 and the annual number of new active TB cases has been decreasing regularly until 2007, then increasing again slightly, mainly because of an increase in cases among foreign-born subjects accounting for 73% of cases in 2014 [16]. In the 1999–2002 period, vulnerable groups of population such as asylum seekers, refugees, undocumented immigrants and homeless persons accounted for 39% of cases in Geneva [17]. In recent years, we observed a change in populations accommodated in shelters with a growing proportion of vulnerable migrants from medium - to high TB prevalence countries, which correlates with findings in other European cities [5]. Unlike Germany where screening for active TB is mandatory for homeless persons living in shelters, Swiss policies restrict systematic screening to asylum seekers. Since January 2006, all asylum seekers undergo a health questionnaire upon entry and active TB prevalence is currently estimated in this group at 124/100′0000 [18]. Before 2006, screening included systematic chest X-ray and tuberculin skin testing. A comparative study showed that the questionnaire was less sensitive but more specific than systematic chest x-ray, with a similar yield in terms of number of cases started on treatment at 90 days, a lower proportion of screenees requiring additional investigations but a longer time-lag before treatment initiation (mean of 6 vs. 25 days) [18]. Screening for latent tuberculosis infection is not considered a priority in Switzerland for this population, because of its high mobility, rendering completion of preventive treatment unlikely, or at least difficult to monitor.

In 2013, we diagnosed two cases of late presentation of active TB in homeless adults living in city shelters in Geneva with several employees working in the shelter showing seroconversion. Moreover, the time-lag between the onset of symptoms and the diagnosis sharply compromised the contact tracing because of the high turnover rate of users in the shelters. There was thus a need to document the local active TB epidemiology in this setting to guide local health policies and strategies.

We designed a program with the social services of Geneva, Switzerland, with the goal of testing a systematic screening program for active TB using the screening tool applied to asylum seekers. Study objectives were to assess 1/ program acceptability in homeless subjects and 2/TB prevalence in this population.

## Methods

### Setting

This prospective study implicated the social services of the City of Geneva and Geneva University Hospitals (HUG), Switzerland. The Geneva City social services operate shelters for homeless persons each winter. Every year, around 1000 people are accommodated, which represents more than 90% of the estimated number of homeless persons in Geneva. During the winter of 2015, 200 beds were available every night, with a peak number of 300 made available in cases of extreme cold. Shelters are underground facilities with 10 to 20-bed rooms. After registration, homeless persons are granted 30 nights free of charge. Stay can be extended according to availability. Shelters also have emergency beds accessible to unregistered persons for a maximum of two consecutive nights. Only name, age and sex of homeless persons are recorded during registration process. Longer stays require full registration at the office.

Access to healthcare is regulated by the purchase of a private health insurance in Switzerland. It is estimated that 1–3% of the population, mostly homeless and migrant persons, are uninsured and thus face difficulties accessing care. HUG is the only public hospital in the Canton of Geneva covering a population of 500′000 inhabitants. While it theoretically provides access to care for all residents in Geneva irrespective of health insurance status, vulnerable groups of population often delay or renounce to healthcare for economic or administrative reasons. Incidence of active TB in Geneva (13.4/100′000) is two times above the national average probably because of a higher proportion of foreign-born residents (40.7% in 2014) [16]. All TB cases in the Canton are managed at the Division of Pulmonary Diseases, HUG, including patients without health insurance.

### Program implementation and management

This program was designed and implemented in collaboration with the Social Services of the City of Geneva. The study board included primary health care physicians, pulmonologists and social workers. Weekly contacts allowed for adapting practices to circumstances and monitoring recruitment and follow up of cases. A one-month pilot phase was conducted the year prior to the present study in order to train social workers and to test the multilingual audio and written information material. It included 115 participants of whom 89 (77.4%) agreed to participate and no active TB cases were found. After testing, audio and written materials were adapted to better fit the field conditions, including increasing the number of languages available.

### Participants

The target population included all persons aged 16 and above registering for shelter accommodation at the Geneva City social services during the shelters activity period from November 1st 2015 to March 30th 2016.

### Recruitment

Recruitment took place at the registration office. First, social workers briefly presented the study during the administrative registration. Then, homeless persons met with the investigators who systematically provided detailed information on risk of TB, screening procedures and incentives using oral, written and pre-recorded audio material in eight languages. After providing written consent, homeless persons completed the screening questionnaire in the language of their choice under the supervision of the investigator. Of note, decision to refuse screening did not impact on access to shelters.

### Screening tool

In order to comply with the national strategy applied to asylum seekers, we used the Swiss Federal Office for Public Health (OFSP) 10-item computer-based questionnaire (www.tb-screen.ch) as a first-line risk assessment. Freely accessible and available in 32 languages, it combines written, visual and audio formats to optimize usability. It also contains a brief information section about TB symptoms and transmission. Questions cover clinical symptoms and exposure to TB and a subjective evaluation of global health status by the patient (3 points) and the investigator (3 points) (Table 1). The total score is strongly modulated by the epidemiological risk based on the participants’ country of origin. Country of origin may yield a score of up to 8 points according to its’ incidence of TB, and up to 10 points if incidence of MDR-TB is 30/10^E5^ inhabitants or more. Scores are calculated according to yearly updates of WHO reports. The maximum score is 26 points. The threshold defining need for subsequent investigation was set at 10 points in accordance with OFSP recommendations and current practice. Smoking status was extracted from the questionnaire (self-reported).

**Table 1:** Items of the tb-screen questionnaire

Item	Points
Country of origin	0 if national TB incidence <20/100′000 1 if 20–49/100′000 2 if 50–99/100′000 3 if 100–149/100′000 4 if 150–199/100′000 5 if 200–299/100′000 6 if 300–399/100′000 7 if 400–499/100′000 8 if ≥500/100′000 or if national MDR TB incidence 20–30/100′000 10 if MDR TB incidence >30/100′000
Currently smoking	1 if positive answer
Cough -If positive answer: Have you been coughing for more than 3 weeks?	4 if positive answer
Cough with phlegm	2 if positive answer
Weight loss over the last 3 months	1 if positive answer
Sweat at night	1 if positive answer
Previous TB treatment	1 if positive answer
TB in member of immediate family	1 if positive answer
Currently feeling sick	3 if positive answer
Impression of poor health by the examiner	3 if positive answer

### Diagnostic procedures

Participants with a score ≥ 10 were informed of the possibility of active TB and of its’ potential risk and transmission. Referral to HUG was organized on site with practical information transmitted orally and on paper. The Division of Pulmonary Diseases Outpatient Clinic received participants during working hours from Monday to Friday without appointment and participants were immediately sent for chest X-rays. TB-trained pulmonologists directly analyzed images and categorized them as indicative or not indicative of active TB. All images were double-checked by radiologists within 48 h. In case of suspicion of active infection, patients underwent the usual TB diagnostic procedures including microbiological testing on spontaneous or induced sputum samples, and when appropriate, bronchoscopy.

### Strategy to enhance retention within the program

We used a combined strategy to enhance retention among participants with positive screening, including repeated reminders by health care workers in the shelters, phone recalls (up to five), delivery of free medical care at HUG and a CHF 20.- (Euro 18.-) shopping voucher for a local supermarket chain given after undergoing the diagnostic investigations.

### Identification of false-negative cases

All patients with pulmonary TB in the Canton of Geneva are treated by the Division of Pulmonary Diseases of Geneva University Hospital. The Canton of Geneva has a centralized mandatory data collection system for all positive cultures for mycobacteria, and HUG is the major treatment center. The Division also systematically enquired about stays in shelters over the previous three months in all incident TB cases in the Canton of Geneva during the recruitment phase and up to three months after its completion in order to confirm that no homeless person refused or failed the screening procedure while in a shelter. Furthermore, treatment of TB also requires mandatory notification. Thus it is highly unlikely that a case of active TB could be missed among persons residing in the Canton.

### Case definition

Homeless persons were either people registering for shelter accommodation or those defining themselves as such during the post study surveillance. Cases were persons registering at the Geneva social services for shelter accommodation during the winter of 2015–2016 with active TB confirmed by PCR and/or positive culture for *Mycobacterium tuberculosis* complex or put on empirical TB treatment. False negative cases were defined as proven active TB pulmonary cases with symptoms beginning while staying in the shelter and negative screening.

### Variable definition

Regions of origin were categorized according to the World Health Organization (WHO) region definition. We categorized countries as having low (0 to 49 per 100′000 inhabitants), medium (50 to 99 per 100′000) or high (100 and more per 100′000) TB incidence according to the most recent WHO data [19]. Homelessness duration referred to the time interval between registration and the last stable accommodation. Due to difficulties to precisely assess this parameter, we categorized as: < 3 months, 3 to 12 months and >12 months. Acceptability was assessed by determining the proportion of eligible participants agreeing to undergo the screening procedure.

### Statistics

We presented categorical data as absolute numbers and proportions, and continuous variables as mean with standard deviation (SD) or median and interquartile range (IQR). To investigate the relation between the variables of interest and possible predictive factors, we used two-by-two tables and chi-square or Fisher’s exact tests for categorical variables and unpaired Student’s t or Kruskal-Wallis tests for continuous variables. Univariate and multivariate logistic regression analysis were used to assess factors associated with screening acceptance. Adjustment was performed for age, sex, health insurance, shelter stay duration and homelessness duration. Data were analyzed using IBM SPSS Statistics (version 23).

## Results

### Population accommodated in the shelters

Overall, 1126 homeless persons aged above 16 years slept in the shelters during the study period. A total of 832 (73.9%) registered with the social service. The 294 who did not register were mostly men (90.6%) with a mean age of 32.4 years (SD: 11) and used the emergency beds for one (IQR: 1) night on average.

### Target population

Data concerning the 832 registered homeless persons are summarized in Table 2. Participants originated from 75 countries with a majority (62.8%) of Europeans. Romania (*n* = 218, 26% of total), France (*n* = 74, 8.9%), Algeria (*n* = 65, 7.8%) and Switzerland (*n* = 50, 6.0%) were the most represented countries of origin. Overall, 61.8% originated from medium to high TB incidence countries. Only 95 (11.4%) had a health insurance in Switzerland. A majority (64%) were without stable accommodation for less than 3 months prior to registration whereas 26% had been homeless for a year or more. On average, people stayed 20 nights in the shelters with a similar proportion of short (<7 days) and long stays (>30 days).

**Table 2:** Characteristics of the target population (*n* = 832) according to acceptance of screening program

	Total population (*n* = 832)	Screening		*p*-value°
		Accepted (*n* = 726)	Refused (*n* = 106)	
	N (%)	N (%)	N (%)	
Male	674 (81)	600 (82.6)	74 (69.8)	0.002
Mean age^a^	38.5 (12.7)	38 (12.2)	42 (11.7)	0.002
Age group^a^ (years)				
< 25	114 (13.7)	111 (15.3)	3 (2.9)	0.001
25–65	686 (82.5)	592 (81.5)	94 (92.2)	0.008
≥ 65	28 (3.3)	23 (3.2)	5 (4.9)	0.364
Origin				
European	522 (62.8)	450 (62)	72 (67.9)	0.282
Eastern Mediterranean	84 (10.1)	74 (10.2)	10 (9.4)	0.866
Africa	185 (22.2)	165 (22.7)	20 (18.9)	0.386
America	26 (3.1)	26 (3.6)	0 (0)	0.065
Asia and Western Pacific	15 (1.8)	11 (1.5)	4 (3.8)	0.112
TB incidence in country of origin				
Low	318 (38.2)	269 (37.1)	49 (46.2)	0.086
Medium	251 (30.2)	225 (31)	26 (24.5)	0.213
High	263 (31.6)	232 (32)	31 (29.2)	0.580
No health insurance in Switzerland	737 (88.6)	650 (89.5)	87 (82.1)	0.032
Homelessness duration (months)^b^				
< 3	507 (64)	453 (66)	54 (57.4)	0.108
3–12	78 (10)	73 (10.6)	5 (5.3)	0.140
> 12	196 (26)	161 (23.4)	35 (37.3)	0.005
Median stay in shelters (nights)^c^	20 (25.3)	19 (25)	27.5 (32)	0.002
Stay duration (nights)^c^				
< 7	203 (26.2)	186 (27.5)	17 (17.3)	0.036
7–30	363 (46.9)	317 (46.9)	46 (46.9)	1.000
> 30	208 (26.9)	173 (25.6)	35 (35.8)	0.038

°Comparison between participants accepting and refusing screening

^a^Available for 828 subjects

^b^available for 781

^c^available for 774

### Program acceptability

Overall, 726/832 (87.3%) registered homeless persons agreed to undergo the screening procedure. The main reasons for refusing were lack of interest (*n* = 39), having already performed TB testing (*n* = 32) or lack of time (*n* = 22). Male gender, age below 25 years, lack of health insurance, homelessness for less than a year, and shelter stay of less than seven nights were associated with acceptance. After adjustment, male gender (OR: 2.14; 95% confidence interval: 1.27–3.62), age below 25 years (OR: 4.16; 95%CI: 1.27–13.64) and shorter homelessness duration (OR: 1.75; 95% CI: 1.06–2.87) remained significant predictors of acceptance (Table 3).

**Table 3:** Factors associated with screening acceptance

	Unadjusted OR (95% CI)	Adjusted^a^ OR (95% CI)
Male	2.06 (1.30–3.25)	2.14 (1.27–3.62)
Age < 25	5.96 (1.86–19.12)	4.16 (1.27–13.64)
Homelessness ≤12 months	1.94 (1.23–3.05)	1.75 (1.06–2.87)
No health insurance	1.87 (1.08–3.24)	1.59 (0.85–3.00)
Shelter stay <7 nights	1.81 (1.04–3.13)	1.63 (0.88–3.00)

^a^Adjustement for all other factors in table

### Results of screening

Scores ranged from 0 to 18 with a median value of 4. Overall, 30 (4.1%) had scores of 10 or more, indicating the need for further investigations. Table 4 compares demographic and clinical data between those with scores below and above threshold value. Of note, we found a high (64.3%) proportion of smokers. Positive screening was associated with originating from a high TB incidence country (*p* = 0.005) with a trend for smokers (*p* = 0.08).

**Table 4:** Characteristics of the participants (*n* = 726) by questionnaire score

	Questionnaire score		*p*-value°
	≥10 points (*n* = 30) N (%)	<10 points (*n* = 696) N (%)	
Male	26 (86.7)	574 (82.5)	0.634
Mean age (year)	38.5 (12.2)	38 (12.8)	0.819
Age groups (year)			
< 25	3 (10)	108 (15.5)	0.460
25–64	27 (90)	565 (81.2)	0.246
≥ 65	0 (0)	23 (3.3)	0.618
Origin			
European	17 (56.7)	433 (62.2)	0.568
Eastern Mediterranean	2 (6.7)	72 (10.3)	0.575
Africa	10 (33.3)	155 (22.3)	0.181
America	0 (0)	26 (3.7)	0.414
Asia and Western Pacific	1 (3.3)	10 (1.4)	0.373
TB incidence in country of origin			
Low	2 (6.7)	267 (38.4)	<0.001
Medium	11 (36.7)	214 (30.7)	0.546
High	17 (56.7)	215 (30.9)	0.005
No health insurance in Switzerland	29 (96.7)	620 (89.1)	0.239
Homelessness duration (months)^a^			
< 3	16 (44.8)	437 (66.4)	0.232
3–12	3 (10.3)	70 (10.6)	1.000
> 12	10 (34.5)	151 (22.9)	0.117
Median stay in shelters (nights)^b^	18 (28)	19 (25)	0.764
Stay duration (nights)^b^			
< 7	8 (27.6)	178 (27.5)	1.000
7–30	13 (44.8)	304 (47)	0.851
> 30	8 (27.6)	165 (25.5)	0.828
Smoker (%)	24 (80)	443 (63.6)	0.080

°Comparison between score groups

^a^Information available for 687 participants

^b^Information available for 676 participants

### Follow-up of participants with positive screening

Among the 30 participants with positive screening referred to the Division of Pulmonary Diseases for further TB diagnostic investigations, 23 (76.7%) came to their consultation within a median time interval of two days (IQR: 4.5). We found no statistically significant factor associated with loss to follow-up.

### Results of diagnostic procedures

After thorough investigations, we found no case of active TB among patients presenting at HUG. However, we diagnosed acute non-TB chest infections in 3 patients.

### Monitoring for false negative cases

Surveillance during and for three months after the study terminated showed that none of the 34 cases with active pulmonary tuberculosis diagnosed in Canton Geneva from November 1st 2015 to June 30th 2016 had stayed in the shelters.

## Discussion

We designed and managed this program in accordance with European recommendations supporting decentralization and intersectoral collaboration [20]. It provided an opportunity for active TB screening for a large majority of homeless persons accommodated in shelters in Geneva, with high participation and retention rates. During the study and in the post-study surveillance, no case of active TB was diagnosed in the target population.

We found a high (87.3%) acceptance rate for the screening procedure, particularly among young males who had been homeless for a limited period of time. Moreover, retention along the diagnostic procedures was higher than expected. This suggests that homeless persons in our area are concerned about health issues and respond positively to opportunities in contrast to the perception of this group having low inclination for preventive health measures [21]. Cooperation with social services, which also provide essential resources such as food, may have enhanced trust towards our program. Incentives may provide a motivational effect and several authors described how appropriate use of incentives enhanced participation to TB screening or treatment programs [22–24]. In Frankfurt, implementing a voluntary X-ray screening program with the help of local social services had a positive impact on participation and coverage [5]. Other studies showed how cooperation between health and social sectors helps to promote health opportunities for underserved groups of population [8, 25]. As opposed to other infectious diseases, involving peer educators does not seem effective in increasing homeless persons participation screening campaigns for active TB. A study in London comparing shelters with and without peers providing health information to residents found no difference in chest x-ray attendance [26]. Yet, we believe that our efforts to increase awareness regarding TB by direct information and involving social workers contributed to the high participation rate. Our findings encourage the implementation of health promoting and preventive activities targeting homeless persons such as influenza immunization or smoking cessation programs [13, 22, 27].

The null TB prevalence confirms the variability of risk among homeless groups. A meta-analysis in Western countries found that prevalence was highly heterogeneous depending on population characteristics, setting and screening strategy [7]. Several reasons may explain our findings: a) a measurement bias due to the lack of sensitivity of the test. The threshold value of 10 for the test was defined and tested by Schneeberger et al. with an acceptable value for sensitivity and specificity, in a population which presently has many epidemiological similarities with our study population [18]. Interestingly, as in our homeless population, 4.1% of asylum seekers in Switzerland tested positive to the questionnaire for a TB prevalence of 0.12%. Therefore modifying the threshold value would not seem appropriate; b) a selection bias by excluding homeless persons not registering for accommodation cannot be excluded. As mentioned above, the surveillance system in place did not detect cases occurring in homeless during and up to 3 months after the study which makes this unlikely; c) the high mobility of participants may have caused acute cases to be declared outside Geneva Canton and thus to not be recorded by the local surveillance system; and d) finally, we postulate that the risk of TB in homeless persons in Geneva differs from that prevailing in other settings because of a different demographic and social profile. Our cohort mostly included young migrants as opposed to older socially excluded autochthonous homeless screened in previous studies [5, 28]. Indeed, in the context of enhanced international movements and restrictive immigration policies in Western Europe, migrants represent a growing share of homeless persons in Western Europe [2, 5]. The profile of homeless people in Geneva reflects this trend with 78% being highly mobile foreigners without residency permit and limited access to care [29]. This finding is unexepected considering that migrants from medium and high-risk countries for TB now represent the population with the highest incidence of active TB in Europe. Further and longer observation periods will allow us to determine if the demographic changes occuring among the homeless population is associated with an increased incidence of active TB. This diversity supports the need to carefully assess the local social and health context and its constant evolution in regards to changes in internationality mobility pattern to define tailored rather than “one-fits-all” strategies.

In other European cities, active case-finding programs based on outreach activities led to a decline in number of cases and case clusters, demonstrating the public health value of such strategies among high risk homeless persons [28, 30]. In the current Geneva context, these results suggest that reinforcing TB health education in shelters, awareness of social workers about symptoms suggestive of TB, facilitating access to care irrespective of legal or insurance status, maintaining close cooperation between social services and reference health centers and close monitoring of the number of homeless among subjects treated for active TB at the reference center may be a reasonable alternative to a systematic screening strategy. Yet, given the constant evolution in migrant populations in Europe, close TB surveillance remains of major importance to rapidly respond to a change in local epidemiology.

Some groups have suggested screening for latent tuberculosis infection (LTBI) and proposed treatment for LTBI when indicated in homeless subjects [24]. In our area however, the homeless population is a very mobile group with a low probability of attending a TB clinic for 4 to 9 months for LTBI. Therefore, focus in our study was on active TB, not on LTBI, and the strategy followed that chosen by the Swiss Federal Office for Public Health for asylum seekers restricting screening to active cases.

Our study has several limitations. First, homelessness encompasses different groups with distinct housing, social and health needs [4]. Therefore, we cannot extend our findings to all people with unmet housing needs in Geneva. Moreover, homeless women were underrepresented and more data are needed to assess their TB risk. Second, we used a questionnaire which although promoted by our national health services, has not been formally prospectively evaluated in a comparative study. In spite of our efforts to identify false negative cases, we cannot exclude that some early clinically active TB cases with negative score at time of screening were missed given their rather short stay in the shelters because of the high mobility of participants. Finally, this study describes point prevalence upon admission in shelters and thus cannot reflect the longitudinal risk of TB in this population.

### Conclusions

Active TB screening in this hard to reach and highly mobile group was well accepted. The null prevalence was unexpected considering the participants’ sociodemographic profile. Our findings highlight the diversity of homeless groups in Europe and the variability of their risk for active TB. It underlines the need for close monitoring of this social group considering the rapid changes in international mobility patterns to tailor preventive and screening strategies to the local context. In our setting, a combination of optimizing access to care and increasing awareness of users and social workers about TB may be currently the most appropriate option.

## Abbreviations

95% CI: 95% confidence interval
HUG: Geneva University Hospitals
IQR: Interquartile range
LTBI: Latent tuberculosis infection
SD: Standard deviation
TB: Uberculosis

## References

[1] Busch-Geertsema V, Benjaminsen L, Filipovič Hrast M, Pleace N (2014). Extrent and profile of homelessness in European memeber states, a statistical update.

[2] Comission E. Confronting homelessness in the European Union. In*.* Edited by Document. CSW. Brussels, European Commission; 2013.

[3] Nordentoft M, Wandall-Holm N (2003). 10 year follow up study of mortality among users of hostels for homeless people in Copenhagen. BMJ.

[4] Fazel S, Geddes JR, Kushel M (2014). The health of homeless people in high-income countries: descriptive epidemiology, health consequences, and clinical and policy recommendations. Lancet.

[5] Goetsch U, Bellinger OK, Buettel KL, Gottschalk R (2012). Tuberculosis among drug users and homeless persons: impact of voluntary X-ray investigation on active case finding. Infection.

[6] Badiaga S, Richet H, Azas P, Zandotti C, Rey F, Charrel R, Benabdelkader el H, Drancourt M, Raoult D, Brouqui P (2009). Contribution of a shelter-based survey for screening respiratory diseases in the homeless. Eur J Publich Health.

[7] Beijer U, Wolf A, Fazel S (2012). Prevalence of tuberculosis, hepatitis C virus, and HIV in homeless people: a systematic review and meta-analysis. Lancet Infect Dis.

[8] Solsona J, Cayla JA, Nadal J, Bedia M, Mata C, Brau J, Maldonado J, Mila C, Alcaide J, Altet N (2001). Screening for tuberculosis upon admission to shelters and free-meal services. Eur J Epidemiol.

[9] Laurenti P, Bruno S, Quaranta G, La Torre G, Cairo AG, Nardella P, Delogu G, Fadda G, Pirronti T, Geraci S (2012). Tuberculosis in sheltered homeless population of Rome: an integrated model of recruitment for risk management. Sci World J.

[10] National Institute for Health and Clinical Excellence: Tuberculosis: active case-finding in underserved groups. NICE guideline no 33. In. Edited by NICE. London, UK,; 2016.

[11] van Hest NA, Aldridge RW, de Vries G, Sandgren A, Hauer B, Hayward A (2014). Arrazola de Onate W, Haas W, Codecasa LR, Cayla JA, et al. Tuberculosis control in big cities and urban risk groups in the European Union: a consensus statement. Euro Surveill.

[12] NICE (2012). Tuberculosis-hard-to-reach groups (PH37).

[13] Craig GM, Zumla A (2015). The social context of tuberculosis treatment in urban risk groups in the United Kingdom: a qualitative interview study. Int J Infect Dis.

[14] Zenner D, Southern J, van Hest R, DeVries G, Stagg HR, Antoine D, Abubakar I (2013). Active case finding for tuberculosis among high-risk groups in low-incidence countries. Int J Tuberc Lung Dis.

[15] Paquette K, Cheng MP, Kadatz MJ, Cook VJ, Chen W, Johnston JC (2014). Chest radiography for active tuberculosis case finding in the homeless: a systematic review and meta-analysis. Int J Tuberc Lung Dis.

[16] Swiss Public Health Federal Office (2015). Tuberculosis in Switzerland in 2014: more multiresistance. Bull OFSP.

[17] Kherad O, Herrmann FR, Zellweger JP, Rochat T, Janssens JP (2009). Clinical presentation, demographics and outcome of tuberculosis (TB) in a low incidence area: a 4-year study in Geneva, Switzerland. BMC Infect Dis.

[18] Schneeberger Geisler S, Helbling P, Zellweger JP, Altpeter ES (2010). Screening for tuberculosis in asylum seekers: comparison of chest radiography with an interview-based system. Int J Tuberc Lung Dis.

[19] Global Health Observatory (2015). World Health Statistics.

[20] Broekmans JF, Migliori GB, Rieder HL, Lees J, Ruutu P, Loddenkemper R, Raviglione MC (2002). European framework for tuberculosis control and elimination in countries with a low incidence. Recommendations of the World Health Organization (WHO), International Union Against Tuberculosis and Lung Disease (IUATLD) and Royal Netherlands Tuberculosis Association (KNCV) Working Group. Eur Resp J.

[21] Baggett TP, Tobey ML, Rigotti NA (2013). Tobacco use among homeless people--addressing the neglected addiction. N Engl J Med.

[22] Tankimovich M (2013). Barriers to and interventions for improved tuberculosis detection and treatment among homeless and immigrant populations: a literature review. J Community Health Nurs.

[23] Lutge EE, Wiysonge CS, Knight SE, Sinclair D, Volmink J. Incentives and enablers to improve adherence in tuberculosis. Cochrane Database Syst Rev. 2015;(9):CD007952. doi:10.1002/14651858.CD007952.pub3. 10.1002/14651858.CD007952.pub3 PMC4563983 26333525

[24] Gupta V, Sugg N, Butners M, Allen-White G, Molnar A (2015). Tuberculosis among the homeless--preventing another outbreak through community action. N Engl J Med.

[25] Harstad I, Henriksen AH, Sagvik E (2014). Collaboration between municipal and specialist public health care in tuberculosis screening in Norway. BMC Health Serv Res.

[26] Aldridge RW, Hayward AC, Hemming S, Possas L, Ferenando G, Garber E, Lipman M, McHugh TD, Story A (2015). Effectiveness of peer educators on the uptake of mobile X-ray tuberculosis screening at homeless hostels: a cluster randomised controlled trial. BMJ Open.

[27] Baggett TP, Chang Y, Singer DE, Porneala BC, Gaeta JM, O'Connell JJ, Rigotti NA (2015). Tobacco-, alcohol-, and drug-attributable deaths and their contribution to mortality disparities in a cohort of homeless adults in Boston. Am J Public Health.

[28] de Vries G, van Hest RA, Richardus JH (2007). Impact of mobile radiographic screening on tuberculosis among drug users and homeless persons. Am J Resp Crit Care Med.

[29] Co G, Evaluanda (2008). Survey of social clubs users in Geneva.

[30] Bernard C, Sougakoff W, Fournier A, Larnaudie S, Antoun F, Robert J, Brossier F, Truffot-Pernot C, Jarlier V, Veziris N (2012). Impact of a 14-year screening programme on tuberculosis transmission among the homeless in Paris. Int J Tuberc Lung Dis.

